# Exploring the effects of noise pollution on physiology and ptilochronology of birds

**DOI:** 10.1371/journal.pone.0305091

**Published:** 2024-06-20

**Authors:** Rida Amjad, Tahira Ruby, Kashif Ali, Muhammad Asad, Ayesha Imtiaz, Samra Masood, Muhammad Qamar Saeed, Maryam Arshad, Saima Talib, Qura-tul Ain Alvi, Afifa Khan, Muhammad Muazim Sharif

**Affiliations:** 1 Faculty of Chemical & Biological Sciences, Department of Zoology, The Islamia University of Bahawalpur, Bahawalpur, Pakistan; 2 Institute of Zoology, Bahauddin Zakariya University, Multan, Pakistan; 3 Department of Zoology, Division of Science and Technology, University of Education, Lahore, Pakistan; 4 Department of Microbiology and Molecular Genetics, Bahauddin Zakariya University, Multan, Pakistan; 5 Department of Zoology, Government Sadiq College Women University, Bahawalpur, Pakistan; Ain Shams University Faculty of Agriculture, EGYPT

## Abstract

Short and long-term sound-induced stress on daily basis can affect the physiology of avian individuals because they are more susceptible to sound stress in an open environment. Objectives: An *ex-situ* study was carried out to determine the impact of noise on physiology and ptilochronology of non-breeding male domesticated quail birds. Methodology: During 60-days long trial, male quail birds, aged 5-weeks, weighing (c.100gm) were used. Out of 72 experimental birds, 18 birds were assigned to the Control Group (G1) while remaining 54 birds were divided equally into 3 treatment groups: Road Traffic noise (G2), Military activity noise (G3) and Human Activities noise (G4). Birds were housed in standard-sized separate cages (20 ×45 × 20 cm), every bird was kept apart in separate cage in open laboratory under maintained environmental conditions. Millet seeds and water were provided to all the experimental birds *ad libitum*. Noise originated from several sources of recorded high-intensity music (1125 Hz/ 90 dB), was administered for 5–6 hours per day. Observations were recorded in the morning and afternoon. The experiment was conducted during the non-breeding season from August to October in triplicate. Blood sampling was done after 60 days. Results: According to the current study, noise stress significantly (p<0.05) increased the concentrations of creatinine, aspartate aminotransferase (AST), alanine aminotransferase (ALT), alkaline phosphatase (ALP), bilirubin, uric acid, cholesterol, triglycerides, total protein, and glucose while a decline in the levels of albumin was seen in treatment birds of G3. While in terms of hematology, total white blood cells count (TWBC), total red blood cells count (TRBC), mean cell volume (MCV) & packed cell volume (PCV) concentrations were raised in blood of treatment birds of G3. In terms of hormones, noise stress significantly (p<0.05) increased the serum concentrations of Corticosterone in G3 while a significant (p<0.05) decline was observed in the concentrations of luteinizing hormone (LH), thyroid stimulating hormone (TSH), and follicle stimulating hormone (FSH) in the same group. Moreover, fault bar formation in G3 was more prominent than others. Conclusion: Noise stress can significantly affect serology, hematology, hormonal physiology and ptilochronology in quail birds.

## 1 Introduction

Noise pollution is one of the numerous new health concerns that are plaguing modern society. Animal life, especially avian life, is significantly impacted by protracted noise [1]. The general physiology of birds is continuously impacted by noise from man-made activities [2,3]. Birds had major issues with mating behaviours, food-seeking behaviours and other negative impacts on physiological and endocrinological alterations as a result of the disturbance in general behaviours [4]. Different types of physiological stress can be seen in animals due to starvation, overcrowding, high density, extreme temperature (cold/hot) and noise. Exposure to these types of stress causes physiological, metabolic and immunological disturbances [5].

According to [6], serum biochemical and hematological markers offer important insights into the immunological state of animals [7]. In a significant part, bird’s hematological profile is a reflection of how healthy they are overall [8]. There is some evidence that noise exposure can have a variety of effects on avian reproductive success. For example, Great Tits, bred in noisy areas, produced fewer fledglings and laid smaller clutches than when nesting in calmer areas [9] and Eastern Bluebirds (*Sialia sialis*) who nest in loud environments also had worse reproductive achievement [10].

Hormones are essential for controlling reproduction and growth in bird species, and their interactions with environmental and reproductive variables can have significant impacts on these processes [11]. Researchers have suggested that exposure to noise stress may cause a reduction in the secretion of GnRH, which may lead to decrease in the release of LH and FSH from the pituitary gland. This decrease in reproductive hormones can have negative impacts on fertility and reproduction [12]. Chronic noise stress has been resulted in a significant (P<0.05) decline in the levels of serum progesterone, estrogen, FSH, and LH hormones in Albino rats [13].

Noise can act as stressor, causing physiological stress reactions such as increased glucocorticoids, which in turn cause reduced immunological functions and elevated oxidative stress [10]. During the prolonged periods of stress, birds may exhibit physical changes that can limit the activity of the HPA axis. At the same time, the bird’s body may produce more glucocorticoids, which are stress hormones that help the body cope with stress. Glucocorticoids, depending on the duration and level of the stress, can have both positive and negative impact on the body [14].

Quails are an appropriate choice of birds for the non-breeding season due to their adaptability to lab conditions. They have a number of benefits, including quick development, early sexual maturity, high egg production rates, simple handling of adults, and a short generation gap [15]. In the current study, we tested whether exposure to noise alone could significantly affect the serology, hematology, hormonal physiology, and ptilochronology of urban dwelling birds by experimentally exposing non-breeding quails to some realistic amounts of various noise sources produced in urban environment.

## 2 Materials and methods

### 2.1 Materials

#### 2.1.1 Animal care

The current treatment trial was analyzed and approved by the Institutional Animal care and Use Committee (285/Zool.) of Islamia University of Bahawalpur.

#### 2.1.2 Study animals

A total of seventy-two male domesticated quail birds (*Coturnix coturnix*) of 3-weeks old with approximate weight (c.70 grams) were purchased from the market, and performed proper conditioning before conducting experiment in order to make sure that physiological indicators may be at par and consistent. The trial was run at animal facility, Department of Zoology, The Islamia University of Bahawalpur for a period of two weeks. The experimental birds being 5-weeks old, weighed around 100gm, were housed in standard-sized cages (20 ×45 × 20 cm), every bird was kept in separate cage of mentioned size in open laboratory under suitable environmental conditions. The temperature was kept stable around 32–35°C^,^ while, humidity was maintained around 25–28%. The light source was also consistent for each group separately having kept in same ambient conditions at the experimental site. The individuals were exposed for around 14 hours to light and 10 hours’ time to dark period to minimize any other stress on birds. At the age of 5 weeks with weight (c.100 grams) the trial started. All the groups were fed with same type and quantity of food. Millet seeds were given to birds three times a day. Out of 72, 18 birds were kept in the control Group (G1) while 54 bird were separated into three treatment groups (18 birds in each group) viz G2 (Traffic noise), G3 (Military noise) and G4 (Human activities noise). The trial was carried out in non-breeding seasons of birds i.e. mid of August to mid of October for a period of 60 days. Millet seeds and water were provided to all the experimental birds *ad libitum*. Birds exhibited no effect on each other during experiment as all groups were kept in separate rooms at same site. Therefore, the noise source of one group did not overlap with other. Daily observations were recorded in the morning and afternoon while for identification purpose, each cage was tagged by using different types of identification marks/IDs/ colored ribbons.

#### 2.1.3 Noise-source categories

Three noise categories were selected: **road traffic source** (motorcycles, automobiles & buses); **military source** (gunfire, explosions & aircraft); and **human activity source** (equipment, alarms, loud speakers, DVD/ CD players).

#### 2.1.4 Application of noise

Noise dose was applied through recorded high intensity music in speakers (1125Hz/ 90 dB) for 5–6 hours daily to three treatment groups (G2, G3 and G4) based on respective noise source for 60 days.

#### 2.1.5 Animal groups

**G1:** All male birds (n = 18) were kept in control group for a period of 60 days.

**G2:** All male birds (n = 18) were exposed to loud traffic noise recordings (90dB, 5–6 hours per day) for a period of 60 days.

**G3:** All male birds (n = 18) were exposed to military noise recordings (90dB, 5–6 hours per day) for a period of 60 days.

**G4:** All male birds (n = 18) were exposed to human activities noise recordings (90dB, 5–6 hours per day) for a period of 60 days.

#### 2.1.6 Blood collection

Blood sampling was done on the 60^th^day of the treatment trial. The birds were euthanized by pricking jugular vein for blood samples from each of the experimental groups. A sum of 18 blood samples from each of the four groups were collected for further analyses (n = 72). The blood samples were collected in EDTA tubes and moved to commercial laboratory for further testing.

#### 2.1.7 Laboratory analysis

After overnight clotting at 4°C, the samples were centrifuged for 20 min at 4,000rpm.

*2*.*1*.*7*.*1 Serum biochemistry analysis*. A clinical chemistry analyzer Olympus AU 640 (Olympus Deutschland GmbH, Hamburg, Germany) was used to determine the concentration of creatinine, albumin, uric acid, cholesterol, triglycerides, bilirubin, total protein, glucose as well as the enzyme activities of ALT, AST, and ALP in the separated serum [16].

*2*.*1*.*7*.*2 Hematological analysis*. Estimation of total red blood cells count (TRBC), packed cell volume (PCV) or hematocrit, mean cell volume (MCV) and total white blood cells count (TWBC) was performed with the aid of an electronic coulter counter (Mind ray cell counter equipment) [17].

*2*.*1*.*7*.*3 Hormonal analysis*. Estimation of gonadotropins (FSH and LH), thyroid stimulating hormone (TSH) and corticosterone (stress hormone) were performed. For hormonal analysis blood sample were centrifuged at 3000rpm for 15 minutes while Sera was stored at -20°C until analysis. All the hormones were analyzed using ELISA technique [13,18].

*2*.*1*.*7*.*4 Fault bars formation phenomenon*. Induced molting was performed in all three treatment groups. Tail feathers were selected to study fault bar formation. Feathers were plucked on the 20^th^ treatment day in alternate manner. Feathers from tail were plucked one after another to induce forced molting [19].

#### 2.1.8 Statistical analysis

For normal distribution of data within groups, we performed homogeneity test by the application of statistical analysis through SPSS version 25. Then performed One-Way ANOVA and Duncan Multiple Range Test with the significance level at (P ≤ 0.05) to avoid any bias in results. Test value (t) and degree of freedom (df) was evaluated by applying independent samples T-test between two groups i.e. Control group compared with respective treatment group. This comparison practice of Control group was repeated with all treatment groups of one parameter, and in all parameters of serum biochemistry, hematology and hormonal results.

## 3 Results

Almost all the parameters analyzed, P value was less than 0.05 but in few cases P value was more than 0.05 (where test value is negative). Degree of freedom (df) was 34 among all groups whereas test value (t) was different, by comparing control group with each treatment group of specific parameters, is mentioned in the interpretation.

A positive test value indicates a significant increase in concentrations while a negative test value indicates a decrease in concentrations of respective groups comparison. It is necessary to mention that negative test value between groups always have non-significant results indicating P > 0.05.

### 3.1 Serum biochemistry

An experimental study was conducted on quail birds to find out the impact of noise stress on serum biochemistry of birds. In this trial, levels of Creatinine, ALT, ALP, AST, Bilirubin, Albumin, Uric Acid, Cholesterol, Triglycerides, Total Protein and Glucose were measured and compared with control group birds. According to findings, creatinine levels among the birds varied significantly across all groups. In particular, G3 (test value/ t = 11.4) was increased significantly when compared to G1 followed by G2 (t = 6.9) and G4 (t = 8.1) (Table 1). ALT levels were also significant among all groups (p<0.05). The mean Alanine transaminase (ALT) concentrations in the birds were significantly higher in G3 (t = 61.2) and G4 (t = 45) followed by G2 (t = 36.9) as compared to G1 (Table 1). The mean Alkaline phosphatase (ALP) concentrations among trial birds varied significantly across all groups. ALP levels showed higher significant levels in G3 (t = 16.4) and G2 (t = 8) followed by G4 (t = 4.8) as compared to G1 (Table 1). The mean serum levels of Aspartate transaminase (AST) among birds of trial varied significantly across all the treated groups while G3 (t = 21.9) and G4 (t = 6.1) showed highly significant results when compared with G1 (Table 1).

**Table 1:** Creatinine, ALT, ALP, AST, Bilirubin, Albumin, Uric Acid, Cholesterol, Triglycerides, Total Protein and Glucose levels of quail birds after exposure to noise stress.

Biochemistry Parameters	Noise Categories (mIU/mL) (Mean±S.E)			
	G1 (Control)	G2 (Traffic)	G3 (Military)	G4 (Human)
**Creatinine**	0.23±0.01^d^	0.39±0.02^b^	0.5±0.02^a^	0.37±0.01^c^
**ALT**	15.15±0.8^c^	16.51±0.42^b^	30.03±0.47^a^	31.27±0.67^a^
**ALP**	48.43±2.5^a^	70.26±1.13^b^	89.54±5.59^a^	62.73±1.69^c^
**AST**	122.56±0.51^d^	173.2±5.95^b^	328.94±9.45^a^	139.62±2.75^c^
**Bilirubin**	0.5±0.02^c^	0.61±0.03^b^	1.07±0.06^a^	0.67±0.03^b^
**Albumin**	1.17±0.02^b^	1.08±0.03^b^	1.87±0.07^a^	0.91±0.05^c^
**Uric Acid**	7.51±0.02^c^	10.92±0.38^b^	12.31±0.21^a^	7.9±0.21^c^
**Cholesterol**	85.68±0.38^c^	112.54±2.71^b^	145.32±2.47^a^	79.41±1.51^d^
**Triglycerides**	144.29±0.03^d^	205.91±12.72^c^	342.13±12.78^a^	256.62±6.14^b^
**Total Protein**	3±1.28^c^	3.41±18.93^b^	3.76±31.01^a^	3.3±0.04^b^
**Glucose**	137.4±1.16^c^	190.87±14.17^b^	308.04±1.98^a^	174.11±1.9^b^

Similar letters (a, b, c, d) in a single row represents non-significant difference between groups according to Duncan Multiple Range Test (P<0.05; df = 34).

In terms of mean serum levels of bilirubin, G3 (t = 0.92) showed significantly higher levels as compared to G1, followed by G2 (t = 0.98) and G4 (t = 0.98) (Table 1). The increased serum albumin concentrations were observed in G3 (t = 9.2), while decreased concentrations were observed in G4 (t = -5.4) and G2 (t = -2.6) when compared with G1 (Table 1). The mean serum uric acid concentration was significant among G1, G2 and G3 groups while concentration of G3 (t = 22.2) were significantly higher followed by G2 (t = 7.9) when compared to G1, while serum uric acid levels of G4 (t = 1.8) did not differ significantly when compared G1 (Table 1).

After noise stress, the mean serum levels of Cholesterol in G3 (t = 23.9) quail birds were significantly higher (p<0.05) as compared to G1, followed by G2 (t = 9.8), whereas G4 (t = -4.0) decreased significantly when compared to G1 (Table 1). The mean serum triglycerides concentrations of G3 (t = 15.4) were significantly higher followed by G2 (t = 4.8) and G4 (t = 18) when compared with G1 (Table 1). The mean serum total protein levels were observed significantly higher (p<0.05) in G3 (t = 10.6) birds when compared to G1 followed by an increase in G4 (t = 6.6) and G2 (t = 4.6) respectively (Table 1). Glucose levels in serum of birds were observed significantly higher (p<0.05) across all groups. Among all groups, G3 (t = 72.3) showed highly significant results whereas, G2 (t = 3.7) and G4 (t = 13.3) showed less significant values for blood glucose levels when compared with G1 (Table 1).

### 3.2 Hematology

The mean TWBC concentrations in blood samples of G3 (t = 10.5) showed significant increase, whereas, G2 (t = 1.7) and G4 (t = 6.8) showed non-significant (p>0.05) results as compared to G1 (Table 2). The mean TRBC counts in G3 (t = 15.0) birds were significantly higher (p<0.05), followed by G2 (t = 9.9) and G4 (t = 2.6) when compared with G1 (Table 2).

**Table 2:** Total white blood cell (TWBC), Total red blood cell (TRBC), packed cell volume (PCV) and mean corpuscular volume (MCV) levels in quail birds after exposure to noise stress.

Hematology Parameters	Noise Categories (Mean±S.E)			
	G1 (Control)	G2 (Traffic)	G3 (Military)	G4 (Human)
**TWBC (10** ^**3**^ **/μL)**	0.04±0^d^	0.12±0^ab^	0.17±0.0^a^	0.07±0^bc^
**TRBC (10** ^**6**^ **/μL)**	2.2±0.01^d^	2.7±0.06^b^	3.2±0.06^a^	2.4±0.11^c^
**MCV (fL)**	65.2±0.26^d^	80.5±0.88^b^	100.2±2.88^a^	74.02±1.18^c^
**PCV (%)**	19.2±0.25^c^	23.4±0.23^ab^	29.7±0.24^a^	23.07±0.22^b^

Similar letters (a, b, c, d) in a single row represents non-significant difference between groups according to Duncan Multiple Range Test (P<0.05; df = 34).

The MCV levels of birds were significantly higher (p<0.05) in G3 (t = 12.1) followed by G2 (t = 22.7) and G4 (t = 7.3) levels, compared to G1 (Table 2). The mean PCV percentage of trial birds from G3 (t = 13.3) showed a significantly higher percentage followed by G2 (t = 12.5) and G4 (t = 11.6) when compared to G1 (Table 2).

### 3.3 Serum hormonal physiology

The serum LH concentrations in blood samples showed significant results among all the treatment groups (p<0.05). LH levels of G3 (t = -2.8) showed a significant decline in hormonal concentrations (p<0.05) followed by G2 (t = -2.4) and G4 (t = -2.1) when compared with G1 (Table 3). Serum FSH levels were significantly different across all treatment groups (p<0.05). FSH levels of G3 (t = -65.3) were significantly lower (p<0.05) when compared to G1 followed by G2 (t = -28.9) while in G4 (t = -4.6) FSH concentrations showed a significantly less decline (Table 3).

**Table 3:** Luteinizing hormone (LH), Follicle stimulating hormone (FSH), Thyroid stimulating hormone (TSH) and Corticosterone levels in quail birds after exposure to noise.

Non- Breeding Trial Hormones	Noise Categories (Mean±S.E)			
	G1 (Control)	G2 (Traffic)	G3 (Military)	G4 (Human)
**LH (ng/ml)**	5.7±0.06^a^	4.7±0.05^c^	4.2±0.04^d^	5.1±0.07^b^
**FSH (ng/mL)**	103.7±0.32^a^	95±27.47^c^	82±23.7^d^	102.4±0.65^b^
**TSH (nmol/L)**	0.51±0.03^a^	0.31±15.9^c^	0.27±0.01^c^	0.39±7.88^b^
**Corticosterone (ng/ml)**	0.6±0.02^c^	0.7±0.01^b^	0.9±0.01^d^	0.8±0.02^a^

Similar letters (a, b, c, d) in a single row represents non-significant difference between groups according to Duncan’s Multiple Range Test (P<0.05; df = 34).

Serum TSH levels of G3 (t = -5.3) showed significantly low levels followed by non-significant (p>0.05) G4 (t = -10.2) and G2 (t = -10.8) levels when compared with control group (Table 3). Corticosterone levels showed significantly higher levels (p<0.05) in G3 (t = 12.5) followed by G4 (t = 13.3) and G2 (t = 7.4) when compared with control group (Table 3).

### 3.4 Ptilochronology

**Fig 1:**
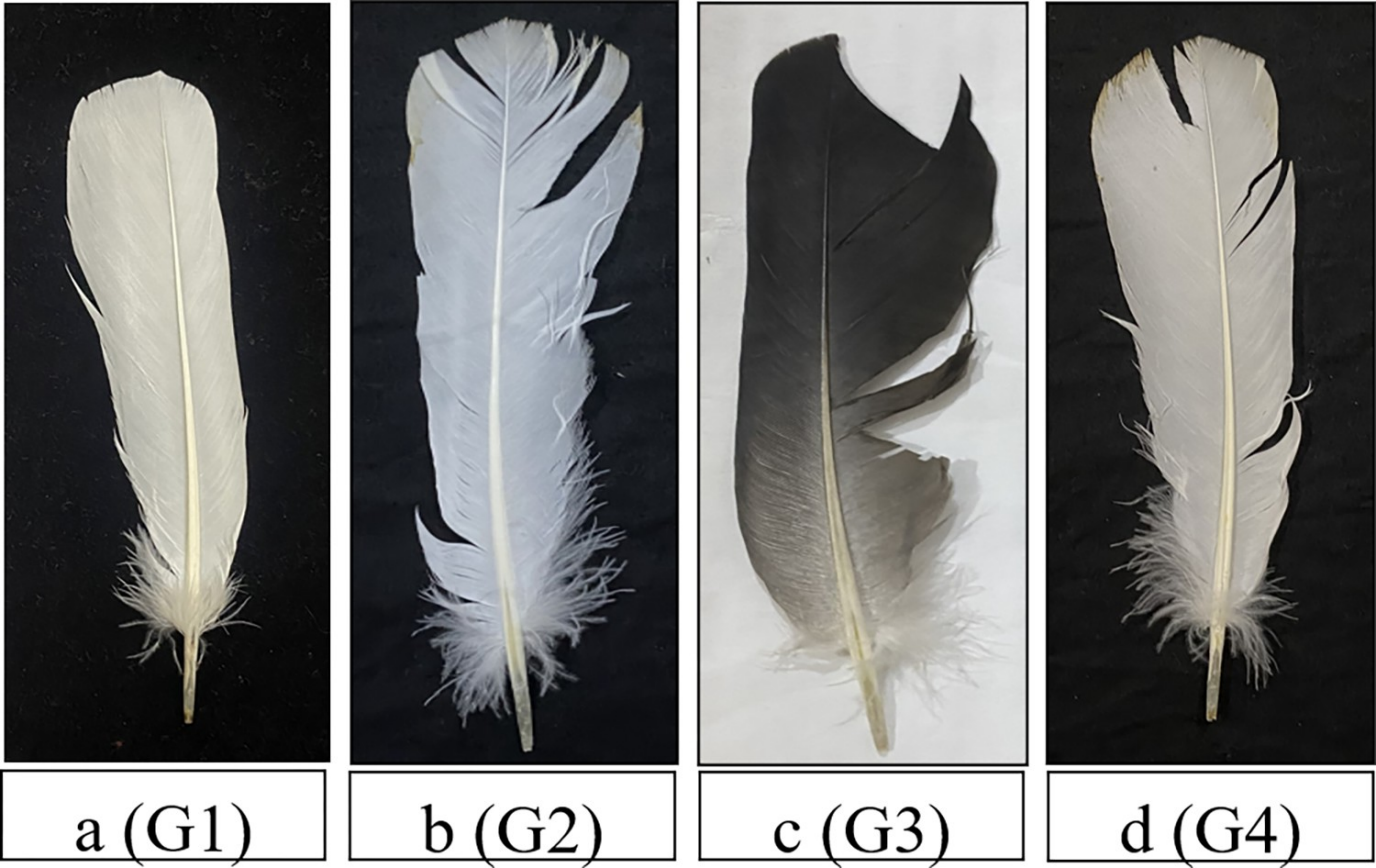
a: G1 (Control Group) showing normal patterns in the feather of non-breeding quail; b: G2 (Traffic Noise Group) showing ptilochronology in non-breeding quail; **c:** G3 (Military Noise Group) showing ptilochronology in non-breeding quail; d: G4 (Human activities Noise Group) showing ptilochronology in non-breeding quail.

Feathers of non-breeding quail birds from G1 have a normal barbule structure of the tail feather. In G2 (traffic noise group) visible fault bars were formed on the right side of the tail feather. On the feathers of non-breeding quails in G3 (military noise group) more visible fault bars were present at the top and right side of tail feather. In the feathers of G4 (human activities noise group) quail birds, less deformed fault bars were seen on top and right side of tail feather (Fig 1).

## 4 Discussion

### 4.1 Serum biochemistry

An experimental trial conducted on Japanese quails in non-breeding season was aimed to check the impact of noise stress on serology, hematology, hormonal physiology and ptilochronology in non-breeding quail birds. The results concluded that different sources of noise stress can affect the overall physiology. In serum biochemistry, levels of creatinine, ALT, ALP, AST, bilirubin, uric acid, cholesterol, triglycerides, total protein and glucose showed increased levels while in hematology, TWBC, TRBC, MCV & PCV percentages followed same increased pattern. Moreover, an increase in the serum hormonal concentrations of FSH, LH, TSH, and corticosterone levels was observed in the quail birds. Fault bar formations in tail feather were also seen.

The above results indicated that an increase in serum creatinine levels was observed when exposed to different sources of noise stress in non-breeding quail. This trend has also been documented by [20] where it was concluded that low glomerular filtration rate ultimately led to an increase in serum creatinine level with the application of noise stress in community of peoples [21]. Results related to liver enzymes are highly positive and are related with the studies conducted by [22] where high levels of noise stress caused an increase in the concentration of liver enzymes in rats. Our results demonstrated that liver enzymes were sensitive to noise exposure leading to a considerable rise in serum concentrations of ALP, AST and ALT. Similar results were demonstrated by [23] in which high levels of liver enzymes were found in rats due to simultaneous noise stress.

Our results revealed a decrease in albumin levels in the serum of birds from chronic noise stress exposure. This corresponds to what was found in another study which showed a rise in blood parameters and a decrease in albumin levels in pekin ducklings when exposed to traffic noise [24]. Furthermore, as shown by our results, it is evident that there was an increase in blood triglyceride and cholesterol levels in response to noise stress. These results are consistent with another study in which malformations occurred in the liver concentrations due to an abnormality between processes of lipid production, consumption, and secretion when exposed to noise stress [25,26]. Additionally, during the course of trial, serum concentrations of uric acid were increased in the birds exposed to noise stress. It is possible to conclude that this increase was due to a high rate of purine breakdown or by the incapacity of the urinary system to remove it [21,27]. The insignificant drop in albumin levels may be related to a decrease in albumen production in the liver as a result of noise exposure. It was described that albumin concentrations were reduced during liver disease [28]. This drop might be attributed to the use of various amino acids in the synthesis of antibodies in response to noise exposure [29].

Our data also showed that noise exposure to birds can cause a highly significant increase in blood glucose levels. In another study in which rabbits exposed to noise displayed an increase in glucogenesis and blood glucose mobilization, which may in turn directly influence various aspects of carbohydrate metabolism [30].

### 4.2 Hematology

As a result of noise stress, there was an elevation in TWBC counts. Similar increase in TWBC counts had been presented earlier in people with psychological stress, as well as in bird models subjected to social stress [31,32]. Noise stress causes nerve fibers to deliver nor-adrenaline into circulation, which sends signals to bone marrow to enhance the creation of hematopoietic stem cells, particularly white blood cells count [33].

The increased number of red blood cells and hematocrit concentrations seen in noise pollution exposure to treatment birds are most likely markers of improved hematopoiesis in bone marrow, as a result of noise-induced stress. However, the physicochemical characters of red blood cells remained constant in terms of shape, mass, and hemoglobin concentration. Long term exposure of noise stress has been shown to cause changes in the hematological and serological characteristics of blood, such as liver damage, reduced antioxidant parameters, and increased enzymatic lipid peroxidation in humans [34–37].

Heat stress lowers hematocrit levels in animals [38]. However, in the current research, treatment groups showed higher mean hematocrit percentages and erythrocyte counts than control group. Increased PCV may be associated with increased metabolic activity needed to satisfy the increased energy demands for development and maintenance during stressful situations [39]. Noise stress can slightly increase MCV in treatment birds, as it was documented earlier in a study of rat serology when they were exposed to high noise stress [40].

### 4.3 Hormonal physiology

Moving on to serum hormonal levels in non-breeding quails, mean serum levels of LH, FSH and TSH were decreased while corticosterone levels increased due to exposure to sound stress. It is obvious that environmental stressors can alter the physiology of endocrine system including metabolism, growth and reproduction. Environmental stress can also affect the secretion of gonadotropin hormones from the pituitary gland which are important for regulating reproductive functions. In reaction to stress, the hypothalamic-pituitary-adrenal (HPA) axis is activated, causing the adrenal gland to secrete stress hormones such as corticosterone. These hormones can suppress the immune system and altering reproductive hormone levelsTop of Form [41].

In accordance with the results of the current study, different studies concluded that glucocorticoid secretions are triggered by stress and cause an increase in secretion from the adrenal gland which eventually results in the reduction of gonadotropins from pituitary [12,42]. With an increase in glucocorticoids, a deactivation of gonadotropin-releasing hormone (GnRH) from hypothalamus occurs (GnRH) [43,44]. The elevated effect of glucocorticoids and the inhibited effect of the pituitary would result in a decline in serum LH levels [45] and FSH levels [12].

According to research, prolonged noise exposure may be linked to thyroid malfunction and increase the incidence of thyroid disorders. The reason is exposure to loud noises can affect thyroid function both directly by altering the anatomy of the thyroid and indirectly by excitatory activation of the hypothalamus [46,47]. Additionally, T3 and TSH blood concentrations dropped during immobilization stress, according to Bianco and his associates [48].

In birds, functioning of thyroid hormones is not controlled by pituitary gland, as in mammals. There are two forms of thyroid hormones present: T3 and T4. From both of these, T4 is responsible for the regulation of TSH, metabolism, fertility and plumage in birds [49]. According to a group of researchers, exposure to intermittent immobilization stress raised corticosterone levels while lowering TSH and growth hormone [50]. When the level of stress hormone corticosterone increased in birds, the inhibitory function of thyroid hormones will start. As a result, a decrease in the T4 occurs [51,52]. Our present results regarding TSH positively correlate with the aforementioned findings, which document that when T4 levels decrease due to stress, TSH levels also decrease as a result of negative feedback in birds [53].

So, research has shown that prolonged noise exposure as a mental stressor can activate sympathetic nervous system (fight-or-flight reactions) as well as cause the production of corticosteroids (response to defeat) when the body is unable to adapt to the noise exposure [46,47,54].

Moreover, it has been shown that the baseline levels of corticosterone levels in animals subjected to persistent stressors are occasionally greater than those of non-stressed animals [55]. During moments of prolonged stress, birds may physically limit activity of the hypothalamic pituitary adrenal (HPA) axis to avoid the pathological consequences associated with persistently increased glucocorticoid concentrations, including weight loss, compromised immunological function and elevated sugar levels [14].

The adenohypophysis secretes corticotrophin (ACTH) in response to loud noises, bright lights, immobility, anxiety, forced exercise, cold, and a variety of other stressful situations. As a result, the plasma levels of ACTH rise, which increases the production of adrenal corticoids. As a result, loud noises increase the plasma levels of corticosterone in rats and “l7-deoxycorticosterone” in humans and monkeys. Furthermore, the rate of aldosterone secretion is accelerated with high ACTH concentrations [56].

### 4.4 Ptilochronology

The study has found that exposure to noise stress caused the birds to experience increased stress levels, as evidenced by physiological measures, and this stress could potentially impact the growth and quality of their feathers. This could have implications for the overall health and fitness of the birds, as well as their ability to survive and reproduce successfully.

Bird plumage is unique because feathers can be grown during a specific molt season and can remain influential throughout a bird’s life. The quality of feathers produced during molt season is dependent on the bird’s overall condition, as energy and nutrients are required to maintain a complete complement of body and flight feathers. Studies have shown that birds under nutritional or any other stress may exhibit reduced feather growth and develop plumage abnormalities as a compensatory mechanism [57–60].

Ptilochronology investigates the link between the rate at which feathers develop and the health of the body by examining if the growth rate of feathers is correlated with the state of the diet while the feathers are development [57,58].

Corticosterone is a glucocorticoid hormone, which means that it is involved in regulating the body’s response to stress. In birds and other animals, corticosterone is secreted by the adrenal glands in response to a variety of stressors, including physical restraint, predator exposure, and food shortage. When birds are exposed to chronic stress, such as food and water deprivation, predator harassment, and environmental disturbance, their levels of corticosterone increase. This can have a range of physiological effects, including inhibiting feather growth and reducing the quality of the feathers that are produced. Thus, chronic stress can have a cascade of effects on bird health and fitness, including affecting their ability to grow and maintain feathers of high quality [61–63].

Based on our results, it seems that fault bars in bird feathers are caused by ecological stress, specifically noise pollution or any other environmental stress. Ptilochronology (the study of growth bars in bird feathers) can be used as a biomarker to assess habitat quality and ecological issues [64,65].

### 4.5 Limitations of study

Our research study suggests a link between noise pollution and fault bars in bird feathers, more research is likely needed to confirm this relationship and to better understand the mechanisms behind it. Additionally, the use of ptilochronology as a biomarker has its own limitations and may not be applicable in all ecological contexts.

## 5 Conclusion

The current study produced some remarkable discoveries about the effect of noise stress on serum biochemistry markers in non-breeding quails. It was found that exposure to noise stress significantly raised the serum concentrations of ALT, ALP, AST, uric acid, cholesterol, total protein and glucose. On the other hand, there was a decline in albumin levels. Additionally, there was a noticeable increase in TWBC, TRBC, PCV and MCV counts in the avian blood samples. The level of physiological gonadotropins (LH & FSH) and TSH in the serum were shown to significantly decline during the non-breeding season. The non-breeding quails also showed a noticeable rise in corticosterone levels. Furthermore, the existence of fault bars in the barbules of feathers was detected in quail birds during the non-breeding season. These findings offer important information about the physiological reactions of domestic male quails to noise stress in non-breeding season.

## Supporting information

S1 File (ZIP)
